# The *slo-1* BK potassium channel interacts genetically with *pmr-1* secretory pathway calcium ATPase during *C. elegans* embryonic cell migration

**DOI:** 10.17912/micropub.biology.000351

**Published:** 2021-01-14

**Authors:** Michellie Thurman, Haonan Sun, Sam Kubica, Vida Praitis

**Affiliations:** 1 Grinnell College, Grinnell IA, 50112 USA

**Table 1. kez8 is a maternal-effect suppressor of pmr-1(ru5ts) lethality and an allele of slo-1:**
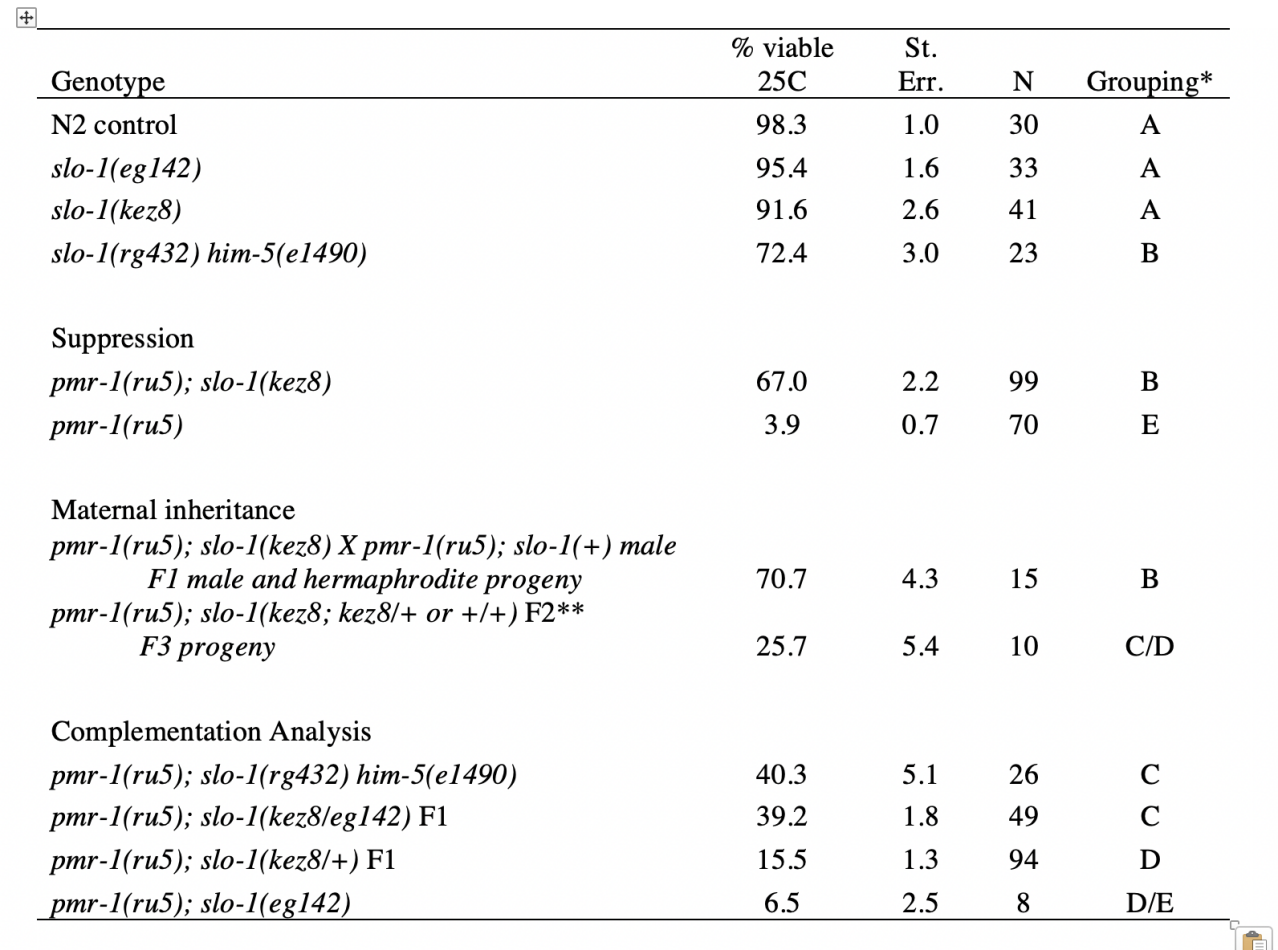
*samples that do not share a letter are significantly different. One-way ANOVA with Tukey comparison p<0.01 (Minitab). ** Chi square analysis of F2 from the cross showed expected ratios for each genotype, with individuals producing viable progeny at frequencies similar to both parental and heterozygous genotypes; p>0.05.

## Description

Calcium signaling is known to play a critical role in cell migration (Ridley **et al.*,* 2003; Wei *et al.*., 2012). In *C. elegans,* embryos with disruptions in the secretory pathway calcium ATPase gene *pmr-1* show defects in cell migration that result in lethal phenotypes. Previous work has shown that *pmr-1(lof)* phenotypes can be suppressed by changes in the activity of the calcium channels IP3-receptor/ITR-1 and ryanodine receptor/UNC-68, indicating that cell migration defects are the results of changes in calcium homeostasis (Praitis **et al.*,* 2013). To identify additional genes important for cell migration during embryogenesis, we performed a genetic screen to isolate additional suppressors of the *pmr-1(ru5)* strain, reasoning that disruption of cell migration due to changes in *pmr-1* activity could be suppressed by commensurate changes in other genes that regulate calcium levels or signaling. In this screen, we identified the *kez8* allele. We confirmed that the *kez8* allele suppresses the *pmr-1(ru5)* mutant phenotype by examining the percentage of viable progeny produced by *pmr-1(ru5); kez8* strains when grown under restrictive conditions. The viability of the *pmr-1(ru5); kez8* strain is 67%, significantly higher than the 3.9% viability observed in the *pmr-1(ru5)* control strain at 25C (Table 1; p<0.01).

Genetic inheritance analysis shows that maternal and not zygotic genotype is crucial for *kez8* suppression of embryonic lethality in *pmr-1(ru5)* strains. *pmr-1(ru5)* hermaphrodites homozygous for the *kez8* suppressor produced viable progeny at similar rates whether they were allowed to self-fertilize or were crossed to *pmr-1(ru5)* males that did not carry a suppressor allele (Table 1; p<0.01). F1 *pmr-1(ru5); kez8/+* hermaphrodites from this cross produced some viable F2 progeny at rates not dissimilar to what would be expected if the allele was acting zygotically (Table 1). If zygotic genotype was critical to F2 survival, we’d predict that individuals homozygous for the *kez8* allele would be over-represented in the F2 population, and these F2 hermaphrodites would produce F3 progeny with viability rates similar to the parental strain. However, when the surviving F2 progeny were allowed to reproduce, the viability of their progeny was significantly lower than the parental strain (Table 1; P<0.01). When we examined the F3 viability rates from F2 individuals, they represented each of the predicted genotypic classes (*kez8, kez8/+* or *+*; ChiSquare, p > 0.05)*,* indicating that zygotic genotype did not affect F2 survival. These results are consistent with a recessive maternal-effect *kez8* suppressor that restores viability to *pmr-1(ru5)* embryos.

Mapping, sequencing, and complementation analysis shows *kez8* is an allele of *slo-1.* We used a whole-genome sequencing (WGS) and mapping strategy to locate the suppressor to the right end of LG V, which was independently confirmed with traditional mapping. Our best candidate for the causative mutation was a single base change, from A to T, in an intron of *slo-1* which maps to position 18.5 Mbp, within ~0.5 Mbp of the highest likelihood map position based on WGS analysis. The mutation was confirmed independently in *pmr-1(ru5); kez8* strainsby PCR amplification and sequencing and is identical to a known *slo-1* allele *rg432*. Complementation analysis with the *slo-1(eg142)* nonsense allele indicates *kez8* is allelic, as *pmr-1(ru5); kez8/eg142* hermaphrodites produce viable progeny at levels that are significantly higher than *pmr-1(ru5); kez8/+* controls (Table 1; p<0.01). However, *pmr-1(ru5)* strains homozygous for *eg142* are not suppressed (Table 1; compare *pmr-1(ru5); slo-1(eg142) to pmr-1(ru5)*; p=1.0) indicating that the unique nature of the *kez8* allele is important for suppression. Neither the *kez8* nor *eg142* alleles show significant embryonic lethality in the wild type background (Table 1). To further support our identification of *kez8* as an allele of *slo-1*, we crossed *pmr-1(ru5)* to a strain homozygous for *slo-1(rg432*), which has the same genetic change in *slo-1* as *kez8* but also carries a linked *him-5(e1490)* allele necessary for the cross but with some associated embryonic lethality in the wild type background (Hodgkin *et al.*, 1979; Table 1). *pmr-1(ru5); slo-1(rg432) him-5(e1490)* hermaphrodites show significant suppression of the *pmr-1(ru5)* embryonic lethal phenotype (Table 1; p<0.01), consistent with our interpretation that *kez8* is an allele of *slo-1*.

The *slo-1(rg432)* allele was identified as a suppressor of a male spicule protraction constitutive phenotype (PrC) caused by disruptions in the *unc-103 and egl-2* EAG-likepotassium channel genes. The *slo-1(rg432)* allele has a significant impact on mRNA expression levels for *slo-1* splice variants in both males and hermaphrodites and reduces the expression levels of other genes that regulate cell excitability in males, including *unc-103* and *egl-2* (LeBoeuf & Garcia, 2012). Our analysis of the *pmr-1(ru5); kez8* strain indicates that the *kez8* allele also negatively impacts male mating. Using *him-14*(dsRNA), which generates a small number of male progeny that can be subsequently crossed to maintain males in a given strain (Fay, 2006), we obtained *pmr-1(ru5); kez8 males*. However, unlike in the parental *pmr-1(ru5)* strain or in other strains undergoing *him-14*(dsRNA) treatment, we were unable to establish stable male lines in the *pmr-1(ru5); kez8* strain, indicating that the *kez8* allele is associated with low male mating efficiency.

SLO-1 is a BK-type calcium-sensitive potassium channel with established roles in *C. elegans* in regulating neurotransmitter release, ethanol intoxication and male mating behavior (Salkoff *et al.*., 2005; LeBoeuf & Garcia, 2012). Our results reveal a new role for *slo-1* in *C. elegans* embryonic cell migration. Given that *slo-1(kez8)*, as well as alleles of other genes identified as genetic interactors of *pmr-1* including IP3-receptor/*itr-1* and ryanodine receptor/*unc-68*, also impact male mating behavior (Garcia *et al.* 2003; Gower *et al.* 2005; LeBoeuf & Garcia, 2012; Praitis *et al.* 2013), a set of similar genes may be involved in both processes. A simple model for the interaction between PMR-1 and SLO-1 is that elevated cytosolic calcium predicted in *pmr-1(lof)* mutants disrupts the normal regulation of BK channel activity during migration, which is suppressed in the *kez8/rg432* mutants because of changes in overall or isoform-specific *slo-1* expression levels (LeBoeuf & Garcia, 2012). The finding that changes in *slo-1* activity impact embryonic cell migration in *C. elegans* is consistent with previously identified roles for SLO/BK-type channels in the migration and invasive properties of glioblastomas (Catacuzzeno **et al.*,* 2015; Ge *et al.*., 2014).

## Methods

We performed a forward genetic screen to identify recessive suppressors of the *pmr-1(ru5)* strain (Praitis *et al.*., 2013) using a MosI transposon mutagenesis protocol (Boulin & Bessereau, 2007). As we could not identify the MosI insertion site, suppressors were mapped using a modified whole genome sequencing (WGS) mapping strategy (Minevich **et al.*,* 2012) where we backcrossed suppressor lines to CB4856 and then used polymorphisms in the Illumina sequenced DNA to locate and identify the causative mutation. The mapped location and sequence change were independently verified using conventional mapping strategies and PCR amplification followed by sequencing. We determined the number of viable progeny produced by single hermaphrodites for all strains, as previously described (Praitis *et al.*., 2013). We used conventional approaches for complementation and inheritance analysis (Brenner, 1974; Fay, 2006; Fay, 2013).

Request a detailed protocol.

## Reagents

We used the following strains: N2, AZ5 *pmr-1(ru5)* I*,* CG925 *slo1(rg432) him-5(e1490)* V, OH441 *otIs45* V, SK4005 *zdIs45* I, BZ142 *slo-1(eg142)* V,CB2065 *dpy-11(e224) unc-76(e911)* V, GRN83 *pmr-1(ru5) I; kez8,* GRN171 *pmr-1(ru5)* *zdIs5* I, GRN414 *pmr-1(ru5); zdIs45 I; slo-1(eg142)* V. Lab generated strains were backcrossed to N2 4 to 6 times. For whole genome sequencing and mapping analysis, lab-generated strains were backcrossed 4 to 6 times to strains with a CB4856 background: GRN229 *pmr-1(ru5) zdIs5* I*,* GRN230 *pmr-1(ru5) zdIs5* I*,* GRN231 *pmr-1(ru5) zdIs5* I*,* GRN232 *pmr-1(ru5) zdIs5* I*,* GRN292 *pmr-1(ru5)* zdIs5 I*; kez8,* GRN293 *pmr-1(ru5)* zdIs5 I*; kez8,* GRN336 *pmr-1(ru5)* zdIs5 I*; kez8.* The strains GRN395 *kez8*, GRN406 *kez8,* and GRN407 *kez8* were derived from parents with CB4856 and N2 backgrounds. All of the GRN strains are from the Praitis lab, as is AZ5. The CG925 strain is from the Garcia lab. Other strains were obtained from the CGC.
